# Treatment outcome and associated factors of patients with atrial fibrillation in adult emergency rooms of selected hospitals of Addis Ababa, Ethiopia: A one-year cross-sectional study

**DOI:** 10.1371/journal.pone.0324626

**Published:** 2025-05-28

**Authors:** Surafel Desalegn, Biruktawit Zemede, Feven Tedla, Tewodros Getnet, Bisrat Tamene Bekele, Eyerusalem Kebede Zewde, Damena mulatu, Teklegiorgis Goshime, Bikis liyew, Kassaye Demeke Altaye

**Affiliations:** 1 Department of Emergency and Critical Care Medicine, School of Medicine, College of Medicine and Health Sciences, University of Gondar, Gondar, Ethiopia; 2 St Paul’s Hospital Millennium Medical College, Department of Emergency Medicine and Critical Care, Addis Ababa, Ethiopia; 3 St Paul’s Hospital Millennium Medical College, Addis Ababa, Ethiopia; 4 Lead catchment hospital, Addis Ababa city administration health bureau, Addis Ababa, Ethiopia; 5 Department of Emergency and Critical Care Nursing, School of Nursing, College of Medicine and Health Sciences, University of Gondar, Gondar, Ethiopia; Kazan State Medical University: Kazanskij gosudarstvennyj medicinskij universitet Ministerstva zdravoohranenia Rossijskoj Federacii, RUSSIAN FEDERATION

## Abstract

**Background:**

Atrial fibrillation is the most common sustained cardiac rhythm disorder with substantial mortality and morbidity from stroke, thromboembolism, heart failure, and impaired quality of life. This study aimed to investigate the emergency presentation, treatment outcome and associated risk factors of atrial fibrillation patients admitted to three tertiary hospitals in Addis Ababa.

**Methods:**

A cross-sectional study was conducted in the adult emergency room of three tertiary hospitals in Addis Ababa. All atrial fibrillation patients admitted to these hospitals from August 15, 2021, to August 15, 2022, fulfilling inclusion criteria were included in the study. Data was collected by reviewing medical records in the form of a structured data abstraction form and analyzed by SPSS.

**Result:**

A total of 133 chart records were reviewed and the mean (±SD) age of study participants was 54.82 ± 20.49 years and ranged between 18–85 years of age. The majority of the patients, 47 (35.3%) were aged between 18–40 years. Nearly more than half of the patients 69 (51.9%) were females and two third [91 (68.4%)] of the patients were from Addis Ababa. Patients of the age group 40–59 with atrial fibrillation have an 82% lower chance of developing stroke than those in the age group >60 years and patients with underlying cardiovascular diseases have seven times higher odds of having a stroke compared to those without underlying cardiac diseases. Male patients with atrial fibrillation had 2.15 times higher odds of staying for > 24 hours at the emergency department compared to female patients. The odds of a > 24-hour stay in the ER significantly increased by 2.7 times as the patient became unstable compared to stable patients.

**Conclusion:**

Males and those with unstable atrial fibrillation have a higher chance of staying more than 24 hours in the emergency room. According to the study, patients who were more than 60 years of age with underlying cardiovascular diseases had a higher likelihood of developing ischemic stroke.

## 1. Introduction

Atrial fibrillation is characterized by either chaotic atrial activity or a total lack of coordinated atrial systole. On the emergency room (ER), this arrhythmia is identified by rapid oscillations of fibrillary waves that vary in size, shape, and timing before each QRS complex [1]. Globally, atrial fibrillation [AF]affects around 30 million people. Significant morbidity and death from stroke and thromboembolic consequences are linked to it [2,3]. Over the past 20 years, the number of instances has increased by 33% [4]. In a study conducted in Cameroon, the community-based prevalence of AF was 4.3% for people over 40 years old and 0.7% for people over 70 years old [5]. There have already been two theories regarding AF mechanisms that were put forth: re-entry, which involves one or more circuits and improved automaticity, which involves one or more foci firing quickly[1]. Atrial fibrillation, most frequently manifests as dyspnea, palpitations, syncope, vertigo, and chest pain are the most typical presentations of AF [6,7]. There are two main goals of treatment; One is preventative therapy to lower the chance of stroke; the other is anti-arrhythmic therapy to ease symptoms [3]. Atrial fibrillation patients on warfarin spent less time in the therapeutic range than was optimal. Furthermore, a poor therapeutic target range was associated with some medications and concurrent heart failure [8]. A study conducted in Canada showed that stroke, mortality, acute coronary syndrome, heart failure, second hospitalization, and electrical cardioversion were the adverse events that were recorded during emergency management of AF and longer length of stay, arrhythmia development, history of stroke or transient ischemic attack, pulmonary congestion, and a non-sinus rhythm at discharge were risk factors for adverse outcomes [9]. Coronary artery disease is a very important risk factor for AF and significantly adds to adverse outcomes in AF patients. Up to 70% of all AF patients have underlying Coronary Artery Disease [CAD] which in recent times has been associated with increased mortality [10,11]. A study done in Ethiopia showed that the prevalence of stroke among AF patients was 19.4%, and patients who had heart failure and thyroid diseases were more likely to have a stroke [12].

Another retrospective study done in Addis Ababa, Ethiopia showed that mitral stenosis, tricuspid regurgitation, age greater than or equal to 50 years, and left atrial size greater than or equal to 45 mm were significant predictors for AF [13]. The study conducted in Cameroon showed that hypertension, followed by valvular heart disease and cardiomyopathy were the primary risk factors for AF, and oral anticoagulant consumption is not ideal [5].

As there is a dearth of information and debates regarding the overall emergency presentations and outcomes of AF, management, and its related complications in developing nations like Ethiopia, as well as the lack of a standard Emergency Room (ER) protocol for management; this study aimed to investigate emergency presentations, and associated factors for treatment outcome of patients with AF.

## 2. Methods and materials

### 2.1. Study setting and design, and period

An institution-based cross-sectional study was conducted by reviewing the charts of admissions made to Saint Paul’s Hospital Millennium Medical College (SPHMMC), Tikur Anbessa Specialized Teaching Hospital (TASH), and Zewditu Memorial Hospital Adult ER from August 15, 2021, through August 15, 2022, with data extraction taking place starting from September 07, 2022 up to October 01,2022.

### 2.2. Participant selection

All adults’ patients aged greater than eighteen who were admitted to adult Emergency Rooms of these hospitals during the study period with a clinical diagnosis and ECG finding of AF were included in the study. Chart records with inadequate documentation were excluded.

### 2.3. Sample size and sampling procedure

Consecutive sampling was used for the study duration August 15, 2021, through August 15, 2022.

### 2.4. Data collection and quality assurance

Before the study, the data extraction tool was prepared from different literature and pretested in 10% of the study sample and corrected accordingly. Pretest was done at another hospital in the Addis Ababa which was no part of the studies sites. The data was collected by trained General practitioners and Nurse and it was check for completeness and aa specific marker on the chart was used to avoid duplications.

### 2.5. Study variables

The study investigates the association between sociodemographic variables, comorbidities, clinical presentation, and complications as independent variables with the in-hospital outcomes of patients presenting to the emergency room as dependent variables.

### 2.6. Operational definitions

#### Atrial fibrillation.

ECG findings on patients’ chart with the absence of discernable P-waves with irregular narrow complex tachycardia.

#### Complications.

Development of heart failure, cerebrovascular accidents related to AF or its treatment (ischemia or hemorrhage) another major bleeding, or drug-related complications.

#### Rate control.

The ventricular rate in AF is below 100

#### Electrical cardioversion.

The joule for synchronized direct current electric shock applied to the patient to achieve conversion to normal sinus rhythm.

#### Emergency management outcome.

Conditions of AF patients in terms of clinical improvement & vital sign stabilization, development of complications, and death.

#### Unstable atrial fibrillation.

Those with systolic blood pressure < 90 mmHg, acute pulmonary edema, rate-related altered mentation, and ischemic chest pain.

### 2.7. Data analysis

After collection, data was cleared, coded, and entered by using Epi Info Version 3.5.3 and exported to SPSS version 20.0.0 for analysis. Data analysis included descriptive statistics, including frequency, percentage, mean, and standard deviation (SD). Crude and adjusted odds ratios were analyzed with a 95% confidence interval (CI) to assess outcomes. Cross-tabulation and chi-squared analysis were done for the categorical variables. Variables with p < 0.25, in bi-variable analysis were entered into multivariable analysis and checked for association with the outcome variables. Factors with P-value <0.05 were declared to have statistically significant associations after multivariate analysis.

### 2.8. Ethical consideration

Ethical approval was obtained from the St. Paul’s Hospital Millennium Medical College Institutional Review Board (IRB) (Ref. No. of pm 21/384). The authors had access to information that could identify individual participants during or after data collection, but names and other personal identities were fully anonymized. The data will be used solely for this research. Since this is a retrospective study of medical records, informed consent was waived by IRB.

## 3. Results

### 3.1. Socio-demographic and clinical characteristics

During the study period, 133 charts (50 from SPHMMC, 56 from TASH, and 27 from Zewditu Memorial Hospital) were retrieved. The mean (±SD) age of study participants was 54.8 ± 20.5 years and ranged between 18–85 years of age. The majority of the patients 47 (35.3%) were aged between 18–40 years. Nearly more than half of the patients 69 (51.9%) were females and two third [91 (68.4%)] of the patients were from Addis Ababa. Fifty (37.6%) of patients were triaged as red patients, 39(29.3%) of the patients were triaged to orange area and, 44 (33.1%) were triaged as yellow and green. The major complaints of the patients during emergency admission were shortness of breath (57.1%), palpitation (11.3%), and body weakness (6%). Around 114 (85.7%) and 73 (54.9%) patients had known underlying cardiovascular diseases and other non-cardiac comorbidities, respectively. Sixty-seven (58.8%) of the patients had a cardiovascular disease with Valvular heart disease. Around 19 (14.3%) of the patients had no underlying cardiac diseases. Furthermore, the most commonly identified comorbidities were Hypertension, Stroke, and Diabetes Mellitus accounting for 44 (60.3%), 20 (27.4%), and 11 (15.1%) respectively. Eighty-five (63.9%) of patients were diagnosed with AF for the first time at the ER while the remaining 48 (36.1%) of patients were known AF patients. Results on socio-demography, clinical characteristics, and diagnosis of AF are presented in the Table 1below. N.B. A single patient could have more than one underlying cardiovascular disease or co-morbidities (**Table 1**).

**Table 1:** Sociodemographic characteristics of AF of AF at SPHMMC, TASH, and Zewditu Memorial Hospital Adult ER, Addis Ababa, Ethiopia, 2022.

Variable		Response	Frequency	Percent
Sex	Male		64	48.1
	Female		69	51.9
Variable	Response		Frequency	Percent
Age in years	Mean ± SD		54.8 ± 20.5	
	18–40 years		47	35.3
	40–49 years		10	7.5
	50–59 years		16	12.0
	60–69 years		13	9.8
	70–79 years		30	22.6
	> or = 80 years		17	12.8
Address	Addis Ababa		91	68.4
	Oromia Region		24	18.0
	Amhara region		12	9.0
	Other regions		6	4.5
The triage category of the patient	Red		50	37.6
	Orange		39	29.3
	Yellow/Green		44	33.1
The major complaint of the patient	Palpitation		15	11.3
	Chest Pain		7	5.3
	Dyspnea		76	57.1
	Leg Swelling		11	8.3
	Syncope		14	10.5
	Body weakness		8	6.0
	Others		2	1.5
Presence of cardiac illness	Yes		114	85.7
	No		19	14.3
Underlying cardiac illness	Coronary artery disease		9	7.9
	Valvular heart disease		67	58.8
	Cardiomyopathy		16	14.0
	Hypertensive heart disease/Hypertrophy		22	19.3
	Pulmonary hypertension		7	6.1
	Congenital heart disease		2	1.8
Presence of Co-morbidities	Yes		73	54.9
	No		60	45.1
Co-morbidities other than cardiac illness	Hypertension		44	60.3
	Diabetes		11	15.1
	Chronic kidney disease		3	4.1
	Thyrotoxicosis		7	9.6
	Stroke		20	27.4
	Asthma		8	11.0
	Other		11	15.1
Diagnosis of AF	New		85	63.9
	Known Diagnosis		48	36.1

### 3.2. Diagnosis and management of atrial fibrillation

**Fig 1:**
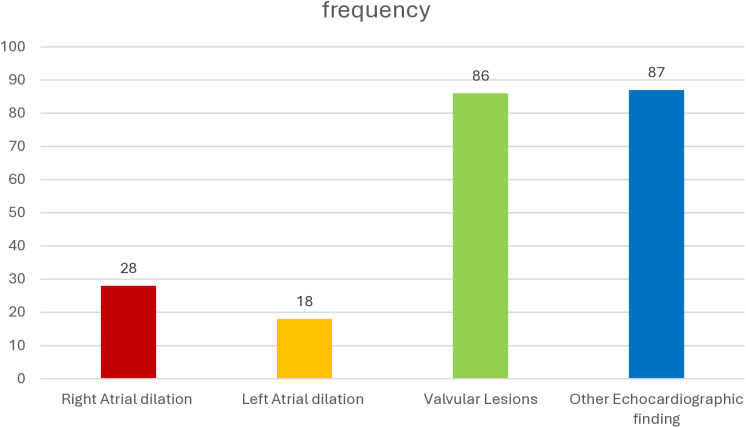
Echocardiographic of AF patients at SPHMMC, TASH, and Zewditu Memorial Hospital Adult ER, Addis Ababa, Ethiopia,2022.

Echocardiography was performed for 107 (80.5%) patients and the majority of the patients 86 (80.4%) had Valvular lesions. Bi-atrial enlargement was found in 30 (28.0%) of the patients (Fig 1).

**Fig 2:**
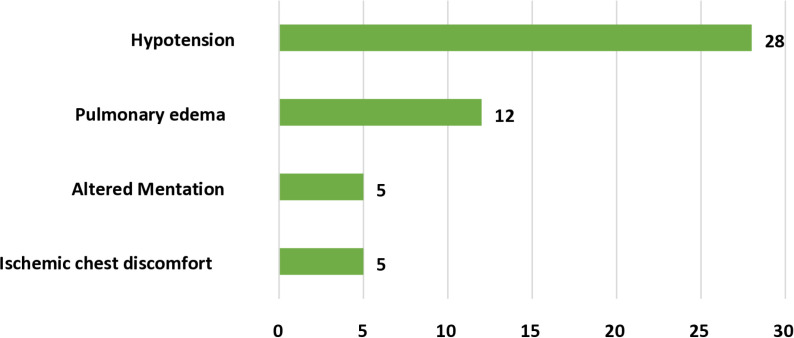
Reasons for physiologic instability of AF at SPHMMC, TASH, and Zewditu Memorial Hospital Adult ER, Addis Ababa, Ethiopia, 2022.

Among the 133 patients who presented in the emergency room 41 (30.8%) of them were clinically unstable due to reasons mentioned in the figure below (Fig 2). Also, pharmacologic rhythm conversion was performed for 8 (6.0%) of patients. 5 (3.8%) of patients needed an emergent electrical cardioversion. Three of the patients needed 100-150J energy and two required > 150J energy for the emergent electrical cardioversion. Only 2 patients were given heparin before electrical cardioversion (Fig 2).

Anti-coagulants, diuretics, and anti-thyroid medications were used in 82 (61.7%), 76 (57.1%), and 3 (2.3%) of patients, respectively. Other medications were being used by 95 (71.4%) of patients and 19 (14.3%) of patients had no history of medication use. Digoxin was utilized in 12 (9.0%) patients in combination with beta-blockers for heart rate control. Among patients on an anticoagulant, 49 (59.8%) had a sub-therapeutic International Normalized rate (INR) range (Table 2).

**Table 2:** Medication and INR range of AF at SPHMMC, TASH, and Zewditu Memorial Hospital Adult ER, Addis Ababa, Ethiopia, 2022.

Variable	Response	Frequency	Percent
Medications the patient was taking for his illness/illnesses	Diuretics	76	57.1
	Anticoagulants	82	61.7
	Anti-thyroid Drugs	3	2.3
	Other medications*	95	71.4
	No medications	19	14.3
Rate control medication the patient was on	Digoxin	76	57.1
	Metoprolol	42	31.6
	Other rate-control medications	5	3.8
	Not on rate control medications	25	18.8
INR range for those on warfarin	Therapeutic	15	11.3
	Sub-therapeutic	49	36.8
	Supra-therapeutic	14	10.5
Variable	Response	Frequency	Percent
	Not determined	55	41.4

*List of other medications statins, anti-hypertensive like Amlodipine, Nifedipine, and Enalapril.

### 3.3. Emergency patient outcomes related to atrial fibrillation

Ischemic stroke was the predominant cerebrovascular accident that occurred in 29 (21.8%) of patients with AF. Fourteen (10.5%) patients had cardiogenic shock. Of the patients treated at the ER, the majority of the patients 63(47.4%) were discharged improved, 65(48.9%) of them were admitted to inpatient services and only one (0.8%) patient died due to cardiac failure.

The average patient duration of stay at the emergency department was 44.3 ± 36.2 hours. Patients stayed at the emergency minimum of 5 hours and a maximum of 10 days. Thirty-five (26.3%) of the patients had stayed for more than 48 hours at the emergency and 64 (48.1%) patients stayed for less than 24 hours (Table 3).

**Table 3:** Outcomes of AF at SPHMMC, TASH, and Zewditu Memorial Hospital Adult ER, Addis Ababa, Ethiopia, 2022.

Variable	Response	Frequency	Percent
Cerebrovascular accidents by the clinical course per se or associated treatments	Yes	29	21.8
	No	104	78.2
The patient goes into cardiogenic shock	Yes	14	10.5
	No	119	89.5
The disposition of the patient	Discharged improved	63	47.4
	Ward admission	57	42.9
	ICU admission	8	6.0
	Death	1	0.8
	Transfer	4	3.0
The patient stays in the ED	Mean ± SD	44.3 ± 36.2	
	6-24 hours	64	48.1
	24-48 hours	34	25.6
	>48 hours	35	26.3

### 3.4. Associated factors for Cerebrovascular Accident in patients with Atrial fibrillation

In bivariate logistic regression, Age, cardiovascular diseases, and INR range were significantly associated with the presence of cerebrovascular accidents. In multivariate analysis, patients of the age group 40–59 have an 82% lower chance of developing stroke than those in the age group >60 years (Adjusted Odds ratio (AOR): 0.179 [95% CI: 0.032,0.991]). Also, patients with underlying cardiovascular diseases have seven times higher odds of having cerebrovascular accidents compared to those without underlying cardiovascular diseases (AOR: 7.021 [95% CI: 1.761,64.801]) (**Table 4**).

**Table 4:** Factors associated with CVA in AF at SPHMMC, TASH, and Zewditu Memorial Hospital Adult ER, Addis Ababa, Ethiopia, 2022.

Variable	Categories		CVA	COR (95% CI)	P-value	AOR (95%CI)	P-value
Yes (%)	No (%)				
Sex	Male	16 (55.2)	48 (46.2)	1.436 (0.628,3.284)	0.391		
	Female	13 (44.8)	56 (53.8)	1			
Age in years	18-39 years	8 (35.3)	39 (37.5)	0.479 (0.187,1.225)	0.124*	0.489 (0.152,1.572)	0.230
	40-59 years	3 (19.5)	23 (22.1)	0.304 (0.081,1.144)	0.078*	0.179 (0.032,0.991)	0.049*
	60 years and above	18 (45.1)	42 (40.4)	1		1	
Address	Addis Ababa	20 (69.0)	71 (68.3)	1.033 (0.425,2.511)	0.943		
	Other regions	9 (31.0)	33 (31.7)	1			
Cardiac illness	Yes	28 (96.6)	86 (82.7)	5.860 (0.748,45.904)	0.092*	7.021 (1.761,64.801)	0.036*
	No	1 (3.4)	18 (17.3)	1		1	
Atrial fibrillation	New	16 (55.2)	69 (66.3)	0.624 (0.270,1.442)	0.270		
	Known	13 (44.8)	35 (33.7)	1			
Atrial dilatation	Yes	5 (22.7)	25 (29.4)	0.706 (0.235,2.122)	0.535		
	No	17 (77.3)	60 (70.6)	1			
Valvular lesions	Yes	17 (77.3)	69 (81.2)	0.788 (0.253,2.455)	0.682		
	No	5 (22.7)	16 (18.8)	1			
INR range	Therapeutic	2 (8.3)	13 (24.1)	0.923 (0.112,7.623)	0.941	1.197 (0.127,11.263)	0.875
	Sub-therapeutic	20 (83.3)	29 (53.7)	4.138 (0.834,20.530)	0.082*	3.606 (0.678,19.176)	0.133
	Supra-therapeutic	2 (8.3)	12 (22.2)	1		1	
	*Significant p-value, COR: Crude Odds Ratio, AOR: Adjusted Odds Ratio						

### 3.5. Associated factors for Emergency Length of Stay in patients with atrial fibrillations

Patient sex, address, and stability were significantly associated with the duration of emergency stay in bivariate logistic regression. In multivariate analysis, male patients had 2.15 times (AOR: 2.150 [95% CI: 1.042, 4.439]) higher odds of staying for > 24 hours at the emergency department compared to female patients. The odds of >24hrs emergency stay significantly increased by 2.7 times as the patient became unstable compared to stable patients with

(AOR: 2.703 [95% CI 1.212, 6.032]) (Table 5).

**Table 5:** Factors associated with the duration of emergency stay of AF Patients at SPHMMC, TASH, and Zewditu Memorial Hospital Adult ER, Addis Ababa, Ethiopia, 2022.

Variables	Categories	Length of stay		COR (95%CI)	P-value	AOR (95%CI)	P-value
>24hrs (%)	≤24hrs (%)				
Sex	Male	39 (56.5)	25 (39.1)	2.028 (1.028,24.051)	0.045*	2.150 (1.042,4.439)	0.038*
	Female	30 (43.5)	39 (60.9)	1		1	
Age in years	18-39 years	23 (33.3)	24 (37.5)	0.784 (0.365,1.686)	0.533		
	40-59 years	13 (18.8)	13 (20.3)	0.818 (0.325,2.057)	0.670		
	60 years and above	33 (47.8)	27 (42.2)	1			
Triage category	Red	26 (37.7)	24 (37.5)	0.989 (0.440,2.226)	0.979		
	Orange	20 (29.0)	19 (29.7)	0.961 (0.406,2.277)	0.928		
	Yellow/Green	23 (33.3)	21 (32.8)	1			
Address	Addis Ababa	51 (73.9)	40 (62.5)	1.700 (0.813,3.556)	0.159*	1.702 (0.785,3.693)	0.178
	Other regions	18 (26.1)	24 (37.5)	1		1	
Non-cardiac comorbidities	Yes	37 (53.6)	36 (56.3)	0.899 (0.454,1.782)	0.761		
	No	32 (46.4)	28 (43.8)	1			
Cardiac illness	Yes	57 (82.6)	57 (89.1)	0.583 (0.214,1.589)	0.292		
	No	12 (17.4)	7 (10.9)	1			
Patient stability	Unstable	27 (39.1)	14 (21.9)	2.296 (1.069,4.933)	0.033*	2.703 (1.212,6.032)	0.015*
	Stable	42 (60.9)	50 (78.1)	1		1	
Cardiogenic shock	Yes	10 (14.5)	4 (6.3)	2.542 (0.755,8.559)	0.332		
	No	59 (85.5)	60 (93.8)	1			

### 3.6. Associated factors for emergency for Emergency Management Outcome of Atrial Fibrillation patients

Bivariate logistic regression showed age, address, non-cardiac comorbidities, and diagnosis of AF, patient stability, and cardiogenic shock as significantly associated with patient outcome. In multivariate analysis, patients in the age group 40–59 years have a 0.077 times lower chance of developing complications than those aged >60 years (AOR: 0.077 [95% CI: 0.018,0.326]). Also, unstable patients have 5.715 times higher odds of complications than stable patients (AOR: 5.715 [95% CI: 1.778, 18.372]) (**Table 6**).

**Table 6:** Factors associated with the pattern of disposition AF patients at SPHMMC, TASH, and Zewditu Memorial Hospital Adult ER, Addis Ababa, Ethiopia, 2022.

Variables	Categories	Disposition		COR (95%CI)	P-value	AOR (95%CI)	P value
Admission/ Transfer (%)	Discharge Improved (%)				
Sex	Male	34 (48.6)	30 (47.6)	1.039 (0.526,2.053)	0.913		
	Female	36 (51.4)	33 (52.4)	1			
Age in years	18-39 years	33 (47.1)	14 (22.2)	1.929 (0.861,4.318)	0.110*	0.872 (0.288,2.644)	0.809
	40-59 years	4 (5.7)	22 (34.9)	0.149 (0.046,0.484)	0.002*	0.077 (0.018,0.329)	0.001*
	60 years and above	33 (47.1)	27 (42.9)	1		1	
Address	Addis Ababa	42 (60.0)	49 (77.8)	0.429 (0.200,0.919)	0.029*	0.558 (0.204,1.525)	0.255
	Other regions	28 (40.0)	14 (22.2)	1		1	
Non-cardiac comorbidities	Yes	34 (48.6)	39 (61.9)	0.581 (0.291,1.161)	0.124*	0.786 (0.302,2.045)	0.786
	No	36 (51.4)	24 (38.1)	1		1	
Cardiac illness	Yes	60 (85.7)	54 (85.7)	1.000 (0.378,2.645)	1.000		
	No	10 (14.3)	9 (14.3)	1			
Atrial fibrillation	New	48 (68.6)	37 (58.7)	1.533 (0.753,3.123)	0.239*	1.503 (0.630,3.586)	0.359
	Known	22 (31.4)	26 (54.2)	1		1	
Patient stability	Unstable	33 (47.1)	8 (12.7)	6.132 (2.549,14.748)	<0.001*	5.715 (1.778,18.372)	0.003*
	Stable	37 (52.9)	55 (87.3)	1		1	
Cardiogenic shock	Yes	13 (18.6)	1 (1.6)	14.140 (1.792,111.557)	0.012*	6.729 (0.500,90.476)	0.150
	No	57 (81.4)	62 (98.4)	1		1	

## 4. Discussion

This study aimed to evaluate emergency management outcomes of AF, associated risk factors, clinical profiles & complications in AF patients admitted to Saint Paul’s Hospital Millennium Medical College, Tikur Anbessa Specialized Teaching Hospital, and Zewditu Memorial Hospital emergency room. Eighty-five (63.9%) of patients were diagnosed with AF for the first time in the ER and the remaining 48 (36.1) patients were known AF patients. This signifies the incidence of AF to be 0.6% during the study period. The study showed the incidence of newly diagnosed AF to be high during the acute illness which is also supported by a systematic review done in Canada which showed the incidence of AF with acute medical illness to be as high as 44% with even higher reports using continuous ECG monitoring [12].

The most common cardiac condition identified in these studies was Valvular heart disease(58.8%) similar to the study done in Addis Ababa, Saint Paul’s Hospital Millennium Medical College showed 59.4% of valvular AF [14]. This is higher than the findings from several recent studies from developing countries that have documented valvular heart disease as a coexisting medical condition in 23.9% to 44% of participants. This might be due to the differences in the epidemiology, study setting, patient population, and smaller sample size in the study [15]. Hypertension, Stroke, and Diabetes Mellitus were the second most common comorbid conditions identified comparable to a similar study done in Massachusetts, USA, which showed the commonest causes of AF being hypertension, ischemic heart disease, and rheumatic valvular heart disease [16]. Contrary to this study hypertension was associated with a lower risk of AF than those without hypertension in a study done in Addis Ababa, Saint Paul’s Millennium Medical College [16].

In this study, the overall commonly prescribed medications were anticoagulants (61.7%) followed by digoxin as a rate control agent (57.1%) which is comparable to a study done in Germany on the incidence and prevalence of AF which revealed commonly prescribed agent as anticoagulant followed by metoprolol and torsemide [2]. The study showed only 19.2% of INR tests to be within the therapeutic range, and the remaining 62.8% and 18.0% of INR values were below and above the expected therapeutic ranges, respectively. This is again supported by a study in one of the anticoagulation clinics of tertiary care hospital in Ethiopia, which showed the number of co-prescribed medications and heart failure was associated with poor time to achieve the therapeutic range with only 12.67% of patients attaining therapeutic range [8]. Lower therapeutic INR values were also reported in a Namibian study (25.2%) [17] and another study in Ethiopia (34.6%) [8]. As opposed to these studies, research conducted elsewhere found higher rates of INR values within therapeutic ranges with a lower percentage of INRs below and above therapeutic ranges [18]. The lower percentage of therapeutic INR might be because the study has a low sample size and nearly half of the patients had no INR reports similar to the aforementioned study could be due to the likely high number of prescribed medications during the emergency stay and plenty of patients presenting with heart failure.

Ischemic stroke was the most common cerebrovascular accident that occurred in 29 (21.8%) patients and 14 (10.5%) of patients had a cardiogenic shock. This finding was coherent with another study done in Academic Canadian hospitals which had found the rate of adverse events within 30 days to be around 10.5% including acute coronary syndrome, heart failure, subsequent admission, or ED electrical cardioversion [9]. Another study done in Addis Ababa, Tikur Anbessa Specialized Hospital, on patients with AF and rheumatic heart disease, showed the prevalence of cardio-embolic events to be lower [9.2%] [15]. This discrepancy might be because this study was conducted in the emergency room while the other study was done in follow-up clinics. Similarly, another study done in Gondar referral hospital showed that there is a five-fold increased risk of developing stroke in heart failure patients with AF [12].

In this study, 63(47.4%) of patients were stabilized in the ER and discharged improved, 65(48.9%) of them were admitted to ICU & wards due to the development of complications, and one (0.8%) patient died of tachyarrhythmia (AF) induced heart failure. The study also demonstrated that being male and having a sign of instability increased the odds of staying for > 24 hours in the ER. This result is different from a study conducted in 6 Canadian hospitals on patients with recent-onset AF and atrial flutter which showed no related deaths and only 1 stroke within 30 days after diagnosis [19]. The absence of death as an adverse event in this prospective study may be due to advanced care for patients with decompensated cardiac failure. In addition to that, the inability to diagnose recent-onset AF in this setting could have resulted in a lead-time bias and misinterpretation of early occurrences of adverse events. The odds of males being prone to prolonged length of stay is statistically significant but other variables which are not included in this study might have affected the results and need further analysis of these variables with a cohort study.

The prevalence of AF with no underlying cardiovascular diseases in this study from all AF patients is around 14.3% which is more or less comparable with a study done on thromboembolic complications in AF by Kopecky et al and another study reproduced comparable results from a hospital-based series by Godtfredsen et al that showed an overall prevalence of lone AF around 0.5%−30%[19). Still, the prevalence of lone AF may be higher than found in this study because the absence of echocardiography in some of the patients might falsely lower the detection of structural heart disease.

Out of the patients presented in the emergency, 41 (30.8%) of them were clinically unstable suggested by vital sign derangement, and pharmacologic rhythm conversion was performed for 8 (6.0%) patients and 5 (3.8%) of patients who needed emergent electrical cardioversion. Only 2 patients were given heparin before cardioversion unlike a study done in 6 academic Canadian hospitals which showed the use of electrical cardioversion reached 97.9% which is similar to these studies about the rare use of heparin as an anticoagulant before cardioversion and no adverse events related to the cardioversion [9].

## 5. Conclusion

Several important discoveries about the emergency treatment of AF are highlighted by this study. Valvular heart disease (VHD) is the most prevalent underlying cardiovascular ailment that causes acute episodes of AF, which primarily affects females and frequently presents in the emergency room (ER) under critical settings. In the emergency room, a lot of patients receive their first diagnosis of AF. Although most of them are given anticoagulants, getting the best possible anticoagulation is still difficult. The increased risk of cerebrovascular accidents in patients over 60 with underlying cardiovascular disease highlights the urgent need for adequate anticoagulation initiation and maintenance. To reduce cerebrovascular accidents, medical facilities are recommended to create methods for preventing VHD and early detection of cardiovascular illnesses. Ensuring standardized AF management in line with guidelines is essential for emergency care professionals.

## 6. Strength and limitations

The study evaluated the different variables related to AF, emergency presentations of patients, and determinants of in-hospital outcomes in such a way that it involved multiple governmental hospitals. This study included all patients with AF treated over the study period which will increase generalizability and decrease information loss. It also focused on emergency patients and showed the acute care perspective of AF patients in some of the largest teaching hospitals in Ethiopia which may help give input for subsequent studies that are going to be conducted in the ER.

This study has several limitations, some of which include; being a hospital-based cross-sectional study, a small patient population, and a heterogeneous study population, which made it difficult to address the temporal relationship between dependent and independent variables. Since the study was done in institutions, it might not be generalized to patients with AF in the general population. Moreover, medical record reports were secondary data with missing information and the absence of important investigations like INR and echocardiography in a proportion of patients. The relationship between the severity of valvular lesions and chamber enlargement was not elucidated. Results from different laboratories taken by different individuals result in measurement bias. Also, there was no way of surely knowing whether AF is newly diagnosed or a recurrence of a previously undiagnosed one. Additionally, since the data is secondary and was not collected for the sole purpose of this study several other variables like smoking, and alcohol which were found to affect other studies were not assessed in this data and, hence, could have affected the outcome of the patients.

## Supporting information

S1 File SPSS file used for analysis. (SAV)

## Abbreviations

AF: atrial fibrillation
CVA: Cerebrovascular accident
ECC: Emergency and critical care
ECG: Electrocardiography
ER: Emergency room
INR: International Normalized Ratio
SPHMMC: Saint Paul’s Hospital Millennium Medical College
TASH: Tikur Anbessa Specialized Teaching Hospital
